# Implementation challenges of India's national anaemia reduction program among pregnant women: insights from mixed-methods implementation research

**DOI:** 10.3389/fgwh.2026.1695442

**Published:** 2026-04-13

**Authors:** Barsha Gadapani Pathak, Pranay Vats, Arun Jadaun, Naveen Garg, Sarmila Mazumder

**Affiliations:** 1 Society for Applied Studies, New Delhi, India; 2 Centre for Intervention Science in Maternal and Child Health, Centre for International Health, Department of Global Public Health and Primary Care, University of Bergen, Bergen, Norway; 3 Palwal Health District Authority, Palwal, India

**Keywords:** anaemia, implementation research, India, mixed method approach, pregnant women

## Abstract

**Introduction:**

Anaemia remains a pressing public health concern in low- and middle-income countries, disproportionately affecting pregnant women. In India, despite longstanding national programs like Anaemia Mukt Bharat (AMB) i.e., “Anaemia free India”. Anaemia prevalence remains alarmingly high with 53% of pregnant women reported to have anemia in India. This percentage is even higher in underperforming districts such as Palwal in Haryana with 56.8% pregnant women being affected by the condition. This implementation research (IR) aimed to identify implementation and contextual barriers to this program across beneficiaries, community health providers, and the health system.

**Methods:**

A convergent parallel mixed-methods design was employed as part of Phase I of this IR nested within the Sustainable Scalable Interventions to Improve Maternal and Newborn Health in India (SIIMA) project. Quantitative data were collected from 370 pregnant women and nine public health facilities through structured assessments and programmatic records. Qualitative data were gathered through 35 in-depth interviews and 6 focus group discussions with pregnant and lactating women, and healthcare providers. The Capability Opportunity Motivation-Behaviour (COM-B) model guided qualitative thematic analysis, supplemented with inductive coding. Integration of findings occurred during the interpretation phase, with qualitative insights used to explain patterns and gaps observed in the quantitative data.

**Results:**

Only 67% of pregnant women received at least one dose of iron and folic acid (IFA), and just 48.6% had undergone haemoglobin testing. Among women for whom both Hb and IFA data were available, merely 5% received IFA dosages aligned with their anaemia severity, as recommended. Mean IFA consumption in the previous month was far below expected levels, while no participant achieved full compliance. Qualitative findings revealed that while both beneficiaries and frontline providers were aware of anaemia and its risks, adherence was undermined by cultural misconceptions, limited counselling, lack of follow-up systems, and fragmented supply chains. Providers cited knowledge adequacy but were constrained by erratic stock availability, diagnostic gaps, and lack of structured supervision.

**Conclusion:**

Despite reasonable awareness and intent, systemic and behavioural challenges impeded AMB implementation. Enhancing supply reliability, strengthening diagnostic, counselling services, and engaging families in decision-making are vital for optimizing program delivery. These findings will inform the development of tailored implementation strategies in Phase II to improve effective coverage and compliance with AMB guidelines in rural India.

## 1 Introduction

Anaemia remains a persistent public health challenge in the developing countries, particularly among pregnant women, despite decades of targeted interventions. Nearly half of all pregnant women in low- and middle-income countries (LMICs) are diagnosed with anaemia, leading to severe maternal and neonatal health consequences. The World Health Organization (WHO) defines anaemia in pregnancy as a haemoglobin (Hb) concentration below 11 g/dL (1), and despite being preventable and treatable, its prevalence remains alarmingly high. According to the WHO dashboard, anaemia affects 53% of women of reproductive age in India, ranking the country fifth highest globally, behind Yemen, Mali, Benin, and Nigeria (2). Within India, Haryana is among the worst-affected states, with 56.5% of pregnant women diagnosed with anaemia (3). At the district level, Palwal continues to reflect a high burden, with 56.8% of pregnant women found to be anaemic (NFHS-5) (4). Despite being a focus district under various national program directed for anaemia reduction, IFA coverage remains suboptimal, only 32% of pregnant women reported consuming IFA for 100 days or more, and a mere 20.5% achieved 180 days of compliance (NFHS-5) (4).

The impact of anaemia extends beyond maternal morbidity, affecting fetal growth, cognitive development, and overall maternal productivity. It is associated with preterm birth, low birth weight, and intrauterine growth restriction, while also contributing to maternal fatigue and reduced work capacity (5). Women in low-resource settings like India face multiple risk factors, including dietary iron deficiency, micronutrient deficiencies, hemoglobinopathies, and infections such as helminthiasis, tuberculosis, HIV, and malaria, leading to a higher burden compared to high-income countries (6).

India has introduced multiple anaemia-control programs since the 1960s, yet prevalence remains unacceptably high. To address this, the Government of India (GoI) launched the Anaemia Mukt Bharat (AMB) program in 2018 under the National Health Mission (NHM) as part of the Intensified National Iron Plus Initiative (NIPI) (7). AMB aims to reduce anaemia prevalence through iron and folic acid (IFA) supplementation, deworming, dietary diversification, and social behaviour change communication (SBCC) (7). Haryana has introduced various strategic interventions, including the Atal Abhiyan (Ensuring Total Anaemia Reduction Initiative) (8). This program is designed to complement the Anaemia Mukt Bharat strategy, aiming for a holistic and systematic approach to reducing anaemia prevalence. Despite these efforts, implementation gaps remain a major barrier to achieving substantial progress. While knowledge among healthcare providers is not a limiting factor, inefficiencies in service delivery, supply chain disruptions, inconsistent monitoring, and low adherence to IFA supplementations persist. Studies suggest the challenge lies not in intervention availability but in their implementation, necessitating context-specific strategies to address health system barriers and community-level adoption (9).

This study was conducted in Palwal district, Haryana, based on a combination of programmatic urgency and contextual relevance. Palwal is among the districts with persistently high anaemia prevalence across all age groups. NFHS-5 data shows that over 50% of pregnant women and children in Palwal are anaemic, exceeding the already high state averages. Moreover, Palwal represents a paradox where agricultural and economic advancement coexist with poor maternal and child health outcomes, underscoring deep-rooted socio-cultural and systemic challenges. Haryana was purposively selected due to its demonstrated political commitment and active implementation of both national (Anaemia Mukt Bharat) and state-level programs, such as Atal Abhiyan and the 100-Day Anaemia Campaign (8). These initiatives indicate the state's proactive stance in tackling anemia as a public health priority. Palwal was chosen in alignment with state-level interest in using locally relevant data to inform program improvements. This setting offered a valuable opportunity to explore the operational dynamics of anaemia control programs in a high-focus district and to generate practical, scalable insights for other underperforming areas across Haryana and similar low-resource settings in India.

## 2 Methodology

This study is part of a broader implementation research (IR) initiative aimed at improving the effective coverage of the Anemia Mukt Bharat (AMB) program among pregnant women in Palwal district, Haryana. The IR project employs a mixed-methods approach, convergent parallel, structured into two phases (10). The current manuscript presents findings from Phase I-the formative research phase, which seeks to examine baseline implementation status and inform the design of optimized intervention strategies. Baseline data were collected cross-sectionally for a period of around two months i.e., from 1st October to 23rd November 2023, prior to the introduction of any intervention components under the IR project. This phase specifically aimed to examine existing service delivery practices and contextual determinants from the perspectives of the health system, service providers, and beneficiaries. The intent was to generate actionable insights to inform the co-design and contextualization of optimized strategies for improved program delivery in Phase II. This IR followed the core-principles of implementation science which have been followed by many Indian studies (11–13).

The study is nested within the ongoing Sustainable Scalable Interventions to Improve Maternal and Newborn Health in India (SIIMA) project, a four-year research initiative (July 2020 to September 2024), which covers a catchment population of approximately 200,000 across 37 villages, divided into nine clusters. For this formative research, clusters 6 to 9, consisting of 19 villages with a population of 64,251, were purposively selected. SIIMA aimed to enhance the quality, continuity, and timeliness of antenatal and postnatal care (ANC/PNC) services and improve access to delivery care across the maternal–newborn health continuum.

Core objectives include (i) identifying key risk factors during pregnancy and their association with adverse maternal and neonatal outcomes, (ii) generating baseline population-level data to inform future intervention testing, and (iii) supporting the development of risk prediction tools using advanced analytics, including machine learning. The remaining clusters (1 to 5) were excluded due to their involvement in a concurrent study titled “Redefining Maternal Anemia in Pregnancy and Postpartum” (ReMAPP) which is a multi-centered, population-based study to establish global haemoglobin thresholds for maternal anaemia. This was done as including these clusters of ReMAPP could introduce programmatic bias through enhanced monitoring. The study area is predominantly rural, with services delivered through six Health and Wellness centers/ Ayushman Arogya Mandir staffed by nine Auxiliary Nurse Midwives (ANMs) and 59 Accredited Social Health Activists (ASHAs).

**Figure 1:**
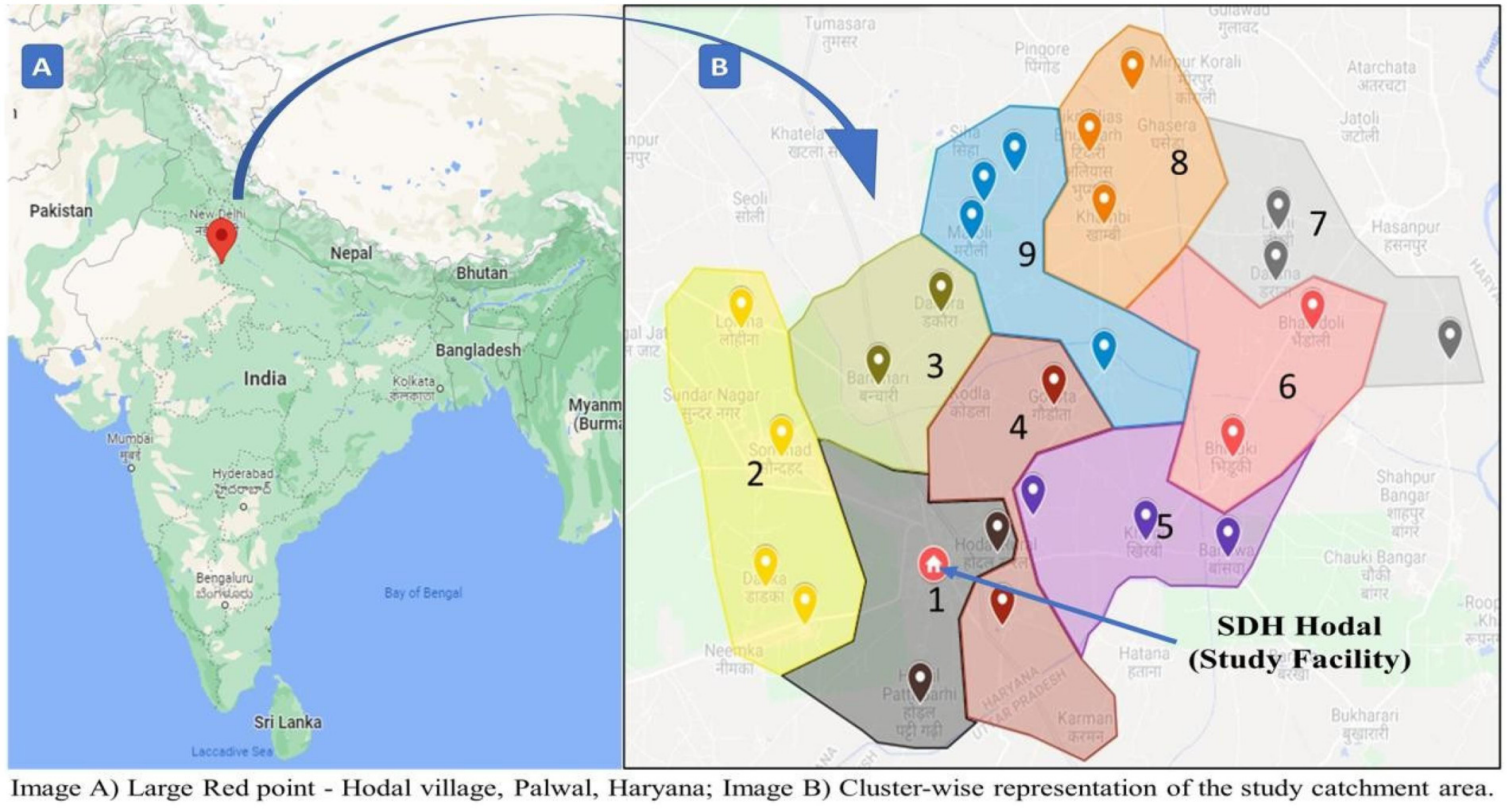
The hodal block (study site) where the implementation research and its phases are being conducted. *Google Maps and MS Paint was used to map out the clusters in the Hodal block*.

The study population included pregnant women in their first trimester, lactating mothers, and a range of healthcare providers at both community and facility levels. These included ASHAs, ANMs, Anganwadi Workers (AWWs), Medical Officers (MOs), Staff Nurses, and Integrated Child Development Services (ICDS) staff. Eligible pregnant women were permanent residents of the study area, identified in their first trimester, and provided informed consent. Women were excluded if they were below 18 years of age, had a nonviable pregnancy (e.g., ectopic or molar), were unable to provide informed consent, or had plans to relocate out of the catchment area during pregnancy or the postpartum period (Figure 1).

To estimate the effective coverage of iron-folic acid (IFA) consumption for at least 100 days during pregnancy, a minimum sample of 310 pregnant women was required at the time of delivery. This assumed 80% expected coverage, ±5% precision, and 95% confidence interval. The calculated sample of 246 was adjusted for a potential non-response rate of 20%, yielding a final target of 310 participants. This sample size was also sufficient to detect a minimum difference of 2.7 g/dL in mean hemoglobin levels from early pregnancy to delivery with 80% statistical power. However, the final number of respondents included in the study was 370 pregnant women. This modest increase was intentional to ensure adequate representation from all four clusters within the Hodal block, proportional to their population size, and to account for potential non-response data.

Pregnant women were identified through an ongoing pregnancy surveillance system under the SIIMA project in clusters 6 to 9 of Hodal block, which covers 19 villages with a population of approximately 64,251. From this population pool, women who were in their first trimester and met eligibility criteria were enrolled using a census-based approach, i.e., all eligible and consenting pregnant women identified in the defined area during the data collection window were approached for inclusion, ensuring representativeness without random sampling (14). Therefore, the final total sample of 370 women was not distributed per cluster but pooled from across the selected clusters. The selection approach was driven by the implementation of the research objectives to generate in-depth, context-specific insights for strategy development rather than population-level generalizability.

Quantitative data were collected through a combination of primary household interviews and facility-level assessments. A structured questionnaire was designed to capture individual-level data on haemoglobin status, receipt and consumption of iron and folic acid (IFA), calcium, and albendazole, as well as compliance and ANC coverage. This tool was administered by trained field investigators to a minimum of 370 pregnant women through direct home visits across selected villages in clusters 6–9. The women were identified through ongoing pregnancy surveillance under the SIIMA project. These structured interviews provided detailed insights into service uptake, supplement adherence, and barriers at the community level. In addition to the interviews, facility assessments were conducted at nine public health facilities, i.e., six sub centres, one primary health centre (Hassanpur), one community health centre (Sondh) and the sub-district hospital. This assessment was conducted to evaluate system readiness, including availability of human resources, diagnostic infrastructure (e.g., haemoglobin testing devices and strips), and medicine stock levels. Relevant secondary programmatic data, such as ANC registers, Health Management Information System (HMIS) reports, and pregnancy surveillance records, were used to triangulate findings and validate self-reported information from the participants. This dual approach was used to comprehensive understanding of both individual behaviours and systemic delivery challenges related to AMB implementation (14). The classification of anaemia was based on severity. The thresholds mentioned in the WHO Guidelines for Haemoglobin cutoffs were used to define each severity category where a woman was classified as anaemic if Hb level is below 11gm/dl, mildly aneamic if Hb is between 10 and 10.9 gm/dl, moderately anaemic if Hb is between 7 and 9.9 gm/dl and severely anaemic if Hb is less than 7 gm/dl.[1] Screening was done using readings from Point of care devices like TrueHb and HemoCue 301+ which draw capillary blood. HemoCue 301+ generally exhibits a higher sensitivity (∼86%) and sensitivity (∼83%) than TrueHb (15). True Hb shows a sensitivity of 78.9% and a specificity of 81%. Combined these devices provide high sensitivity and specificity in anemia detection in resource hit settings, supporting their use as reliable screening devices (15).

To explore the contextual and behavioural determinants influencing AMB implementation, a qualitative component was undertaken using in-depth interviews (IDIs) and focus group discussions (FGDs) across a range of stakeholders (16). A total of 35 IDIs and 6 FGDs were conducted between October and November 2023 in the selected clusters of Palwal district. Participants were selected using purposive maximum variation sampling to ensure a broad representation of perspectives. The IDIs included 5 Accredited Social Health Activists (ASHAs), 5 Auxiliary Nurse Midwives (ANMs), 5 Medical Officers (MOs), 5 staff nurses, 1 Child Development Project Officer (CDPO), 7 pregnant women (primarily in their second trimester), and 7 lactating women (with children under six months of age). FGDs were conducted separately with pregnant women (*n* = 2), lactating mothers (*n* = 2), ASHAs and Anganwadi Workers (*n* = 1), and ANMs (*n* = 1). The inclusion of both pregnant and recently delivered women allowed for an in-depth understanding of service experiences across the continuum of care, from antenatal to postnatal phases. While first-trimester women were prioritized for quantitative data collection to assess early uptake of services, lactating mothers were included in the qualitative sample to capture retrospective insights into adherence, counselling quality, and system responsiveness. Semi-structured interview guides were developed based on the Capability, Opportunity, Motivation–Behaviour (COM-B) model and adapted to local programmatic contexts (17). All guides were translated to local language, Hindi and interviews were conducted in Hindi by trained field researchers, audio-recorded with informed consent, and transcribed verbatim for analysis.

### 2.1 Operational definitions

#### 2.1.1 Insufficient quantities for medicine stock

This refer to IFA stocks that were inadequate to meet the estimated requirement based on the number of pregnant women currently registered at each facility and the prevailing anaemia prevalence in their catchment areas. The estimated demand was calculated by multiplying the number of pregnant women with the recommended daily dosage and duration of IFA supplementation as per AMB guidelines, adjusted for local anaemia prevalence.

#### 2.1.2 Medicine compliance

The extent to which a pregnant woman consumed iron and folic acid (IFA) tablets as per the prescribed dosage and duration recommended under the Anaemia Mukt Bharat guidelines. For anaemic women (Hb < 11 g/dL), the recommended dose is two IFA tablets per day, amounting to 60 tablets over a 30-day period. Therefore, full compliance was defined as consuming 100% (60 tablets) of the required dose in the past 30 days.

### 3.1 Data analysis

#### 3.2.1 Quantitative analysis

Descriptive statistics including means, standard deviations, medians, and proportions were calculated using SPSS version 26.0. Programmatic and facility data were triangulated with ANC registers, HMIS records, and pregnancy surveillance data from the SIIMA platform. Cleaned datasets were used to generate monthly progress updates and inform implementation readiness.

#### 3.2.2 Qualitative analysis

Thematic analysis was conducted using NVivo 12 software, following Braun and Clarke's six-phase approach or framework (18). A hybrid analytical strategy was employed that integrated both deductive and inductive approaches. Deductive coding was informed by the Capability, Opportunity, Motivation–Behaviour (COM-B) model to systematically examine behavioural determinants of program uptake and compliance (17, 19). This framework guided the initial categorization of codes relating to knowledge, access, motivation, and environmental enablers or constraints for both beneficiaries and providers (19). As the analysis progressed, an inductive approach was used to identify themes that extended beyond the predefined COM-B domains, such as program fragility, stock inconsistencies, trust deficits, and gendered barriers to care-seeking, allowing for a more grounded and nuanced understanding of contextual implementation challenges. Transcripts were independently coded by two researchers trained in qualitative methods. An initial inter-rater agreement of 89% was achieved, and coding discrepancies were resolved through iterative discussions and consensus meetings (20). A senior qualitative researcher was consulted when consensus could not be reached (20). To enhance validity, triangulation was conducted across multiple respondent types (pregnant/lactating women, ASHAs, ANMs, MO, staff nurses, ICDS staff) (21, 22). Additionally, member checking was undertaken with a subset of participants to ensure the authenticity and trustworthiness of interpretations (23).

## 4 Results

Although all eight public health facilities in the catchment area reported having stock of iron and folic acid (IFA) tablets, six out of eight facilities (75%) had insufficient quantities. Similarly, there were inconsistencies in diagnostic preparedness. While CHC and PHC facilities used HemoCue 301™, all Health and Wellness Centres (HWCs) used TrueHb™ for hemoglobin (Hb) testing.

However, only 50% (four out of eight) of the facilities had both Hb diagnostic devices functional, and just three of those had sufficient Hb testing strips in stock at the time of assessment. In some cases, even when diagnostic equipment was functional, the unavailability or expiration of test strips rendered them unusable (Table 1).

**Table 1:** Status of facilities in the catchment area on supplies related to anaemia mukt bharat program.

Facility Name	January 2024			
	Current IFA Stock (No. of Pills)	No. of Pregnant Women served under the facility	Hb Apparatus	Hb strips
CHC Sondh	34,800	5,577	Two HemoCue apparatus are available and functional	925
PHC (1)	2,200 (insufficient)	2,751	Two HemoCue apparatus available and functional	1,000
HWC (1)	440 (Insufficient)	427	One of the two Hb testing kit functional	50
HWC (2)	150 (Insufficient)	398	Not available at facility, one available that ANM used to keep with herself	1,800
HWC (3)	500 (Insufficient)	387	Two TrueHb devices are available and functional	0
HWC (4)	50 (Insufficient)	551	Two TrueHb devices are available and functional	1,500
HWC (5)	500 (insufficient)	325	One functional one not functional	70
HWC (6)	1,100	276	Not available	0 (Only expired strips available)

A facility was assessed to have sufficient IFA stock if they had enough no. of pills to fulfill the daily dose requirement of all pregnant women under that facility and considering ∼60% of them are anemic and require a daily double dose.

Sociodemographic profiling of the population in clusters 6 through 9 showed that the majority of the households (79%) fell within the above poverty line (APL) category, and women of reproductive age had a mean of 10.9 years of formal education, suggesting relatively high baseline literacy. Despite this, significant service delivery gaps were evident in AMB coverage (Table 2).

**Table 2:** Sociodemographic data of households in catchment area.

Indicator	Number (%)
Villages	19
Population	60,562
Number of Households	10,726
APL/BPL card available [*N* = 8,946]	7,067 (79)/1,879 (21)
Female-male composition	28,666 (47.3%)/31,886 (52.6%)
Education (mean years of formal education)	10.9 years
[Women (18–49 years age)]	

*APL: Above poverty line; BPL: Below poverty line.

**Figure 2:**
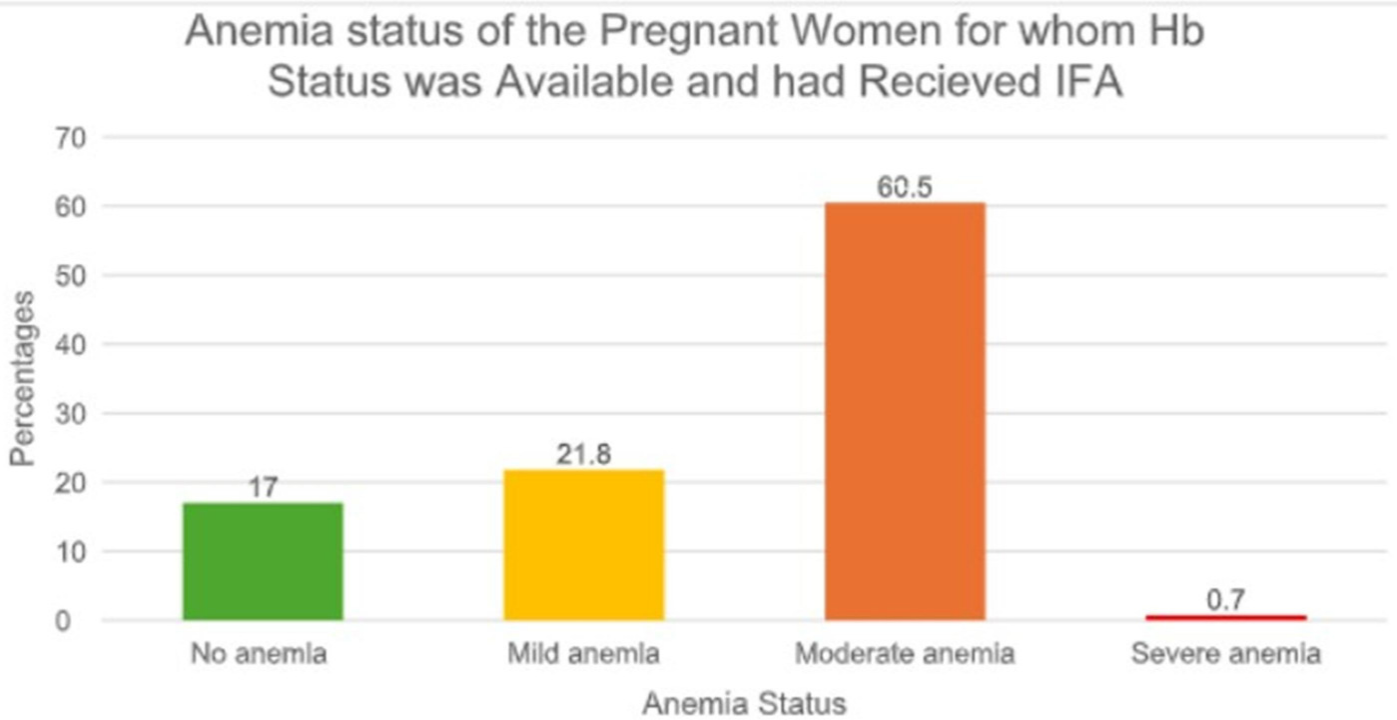
Distribution of anaemia severity among pregnant women with available haemoglobin status and who received IFA supplementation (*n* = 147/370).

Anaemia was highly prevalent among the subgroup of women with known Hb status. Figure 2 shows the distribution of anaemia severity among the 147 pregnant women (40% of the total sample) who had both haemoglobin data and records of IFA receipt. Moderate anaemia was the most prevalent, affecting 89 women (60.5%), followed by mild anaemia (32 women; 21.8%). Severe anaemia was identified in only one woman (0.7%), while 25 women (17%) were non-anaemic. This pattern underscores the predominance of moderate anaemia and highlights persistent gaps between diagnostic coverage and treatment adequacy under the Anaemia Mukt Bharat programme.

At baseline, the mean number of IFA tablets received by the 147 women with available Hb data was 22.4 ± 16.2 tablets, while the mean number consumed in the preceding 30 days was 12 ± 9.8 tablets. The median values (received = 20, consumed = 10) suggest that although most women received a standard 30-day pack, many consumed fewer. Compliance with the recommended regimen was suboptimal—42.2% (*n* = 62) had taken at least 25% of the required tablets, 10.9% (*n* = 16) had taken ≥50%, and none achieved full (≥95%) adherence. Only 5% (*n* = 9) had received IFA dosing appropriately to their anaemia status as per AMB guidelines. Compliance categories were cumulative and not mutually exclusive (e.g., women meeting 50% adherence were also counted under the 25% threshold) (Table 3).

**Table 3:** Baseline status of IFA coverage, haemoglobin testing, and compliance Among pregnant women in the study area (October–November 2023).

Indicator	Baseline (Oct 9th, 2023, to Nov 1st, 2023) N (%)
Number of PW in the catchment area	370
Number of PW who received IFA	248 (67)
Number of PWs with Hb status available	180 (48.6)
No. of PW among the received for whom Hb data is available	147 (59.3)
Number of PWs with Hb status and receiving appropriate number of IFA	9 (5)
**Details of IFA received**	
Mean no. of IFA received (SD) in last 30 days	22.4 (SD 16.18)
Median no. of IFA received (IQR) in last 30 days	20 (20,20)
**Details of IFA Consumed**	
Mean no. of IFA consumed (SD) in last 30 days	12 (SD 9.8)
Median no. of consumed (IQR) in last 30 days	10 (4,20)
**Level of compliance**	
Full compliance	0
>95% compliance	0
>75% compliance	1
>50% compliance	16 (10.9%)
>25% compliance	62 (42.2%)

### 4.1 Qualitative findings from the beneficiaries (*pregnant and lactating mother*)

The qualitative findings analyzed using a mixed inductive–deductive approach guided by the COM-B framework from data on experiences of pregnant and lactating women in Palwal district. The findings revealed a complex interplay of capability, opportunity, and motivation, each influencing their behavior and participation in the Anemia Mukt Bharat (AMB) program.

Themes from the deductive COM-B model were complemented by inductively derived health-system factors, reflecting the contextual realities of implementation in Palwal. These insights highlight why, despite being aware of the program and receiving occasional supplements, adherence and utilization remain suboptimal (Table 4).

**Table 4:** Qualitative findings using mixed analysis i.e., inductive and deductive (COM-B) model to summarize the findings from lactating and pregnant women.

Theme	Subtheme	Verbatim
	Awareness about IFA supplementation	You should take strength-giving pills, like iron tablets.
	Understanding of diagnostic practices	They said everything is fine just by checking my eyes and tongue … . so I think this is how doctors get to know (Pregnant woman)
	Understanding dietary practices during pregnancy	One should eat fruits, vegetables, salads, and drink milk … … ANM gave me good idea when I asked her (Pregnant woman)
	Awareness of ANC visits and check-ups	Whenever there is any issue in the body, we should consult a doctor … i don’t go to doctor without any body issues
	Use and interpretation of MCP cards	*Nothing is written in the vaccination card; it's only used for vaccines*. (Pregnant woman)
	Understanding food safety and hygiene	*Washing vegetables before cutting helps retain nutrients* … … also *is a good practice as we will not fall sick* (Pregnant woman)
	Perception of healthy diet during pregnancy	*Fruits and milk should be consumed, along with strength-giving pills* … . some of these pills are available in our sub-centers (**Pregnant woman**)
Opportunity	Availability of IFA supplements	*I was given two strips: one of calcium and one of iron from the health facility.* (**Pregnant woman**)
	Access to healthcare facilities	*Vehicles run; they are available at the corner, and charge 30 rupees* … … *so this is more money when my husband is not working and I don't prefer to use it unless it is very much important for my child's (un- born) and my health* (**Pregnant woman**)
	Delayed care due to household roles	*No one was available to accompany me; my husband was at work* … *. And I have so manh jobs at home* … . *even the children's care I have take*. (Pregnant woman)
	Role of community level health care staff	*The AWW called us for a meeting, but no one was there * … … . *it was already difficult to reach the AWC and we didn't receive any information (***Pregnant woman)**
	Challenges in receiving AMB program services	Only one injection was available at the facility; I took the remaining five from a nearby doctor.
	Availability of dietary supplements	Only one packet (milk powder) was given, and it was lumpy … didn't like it (**Pregnant woman)**
	Stock-outs and supply chain issues	*During my daughter's time, the doctor said blood was low but we didn't receive any tablets*. (**lactating woman)**
	Gaps in counselling and IEC activities	*No one told us anything after delivery * … … . *I asked the ASHA and ANM one day* … . *But they were not providing any relevant information*’ **Lactating woman)** No one said anything after delivery … … . I always want to know what is good for child and me … .
Capability	**Knowledge of:**	
	Anaemia and prevention	*“If haemoglobin is below 9* … . *I think* … *it means that my blood is low”.*
	Anemia symptoms	*Blood becomes watery, and there is dizziness*. (**Pregnant woman**)
		- *Swelling appears in the legs and body* … . *then it may be blood less in the body ‘*
		-*’Weakness happens; the body seems like it's falling apart* … *. I have no energy to do anything for me or my house’*
	Quality of ICDS services	*I will be happy to take the food from centers but most of the food they give are not good quality and I don't consume*. (**Pregnant woman**)
Motivation	Perceived side effects of IFA supplements	*The small red pill made me vomit* … … . I prefer throwing it then consuming it ‘**Pregnant woman**)
	Cultural practices influencing diet	*Ghee should be consumed; it helps the baby grow*.
	Perceived lack of benefit from supplements	*Didn't feel any benefit or harm* … . *I feel there is no use of consuming such medicines (***Pregnant woman)**
	Compliance with ANC visits	*I am fine going to ANC visits as they do tests and consultation well like my Ultrasound was normal, the doctor checked my eyes and tongue and said haemoglobin is fine* … *. all these made me feel good (***Pregnant woman)**
	Cultural food taboos	*In the last month I reduced eating; if the baby grows big, it will be hard to deliver.* (**Pregnant woman)**
	Lack of follow-up guidance	*Doctor said blood is low but didn't tell me anything else* … . *so I didn't know what to do and was not feeling any need to go for further check-up* (**Pregnant woman**)
	Family influence on healthcare decisions	*My mother told me not to eat sour food; it causes the stitches to get infected* … . *So I listened to her as she knows everything.* (**Lactating mother)**
	Over-reliance on private sector	*We went to Kamlesh Hospital after a few days; they did not say anything* … . *they know everything and always give good advice* … . *so we don't worry* (**Pregnant woman)**
	Burden of household work	*I haven't even eaten yet; these children don't give me time* … . *I always feel stressed and sad as I am not giving much time to look after myself* (**Pregnant woman)**
***Emergent Health-System Factors (Inductive Themes)***		
Health systems and healthcare issues	Healthcare provider attitudes	She is rude and doesn't say anything.
	Stock-outs and supply chain issues	During my daughter's time, the doctor said blood was low, but we didn't receive any tablets.
	Service-delivery gaps	No one told us anything after delivery … … its very difficult to do things without any knowledge as I don't have my mother-in-law at house (**lactating woman**)
	Waiting time	Long queue was there; my number never came.
	Social determinants of health	There were financial issues once or twice, but we had to manage for the baby's sake.

#### 4.1.1 Capability: knowledge, awareness, and understanding

Most women demonstrated only basic awareness of anemia, often describing it as “low blood” or “watery blood,” and associated it with fatigue, dizziness, and swelling. One pregnant woman said, “*Blood becomes watery, and there is dizziness.*” However, few understood the clinical classifications or severity levels. Their understanding of diagnostic practices was also limited, with many if doctors could assess anaemia by “looking at eyes and tongue,” as one respondent shared, “*They said everything is fine just by checking my eyes and tongue … I think this is how doctors get to know*”*.*

Awareness about IFA was framed around strength or “energy pills,” and while many recalled receiving strips of tablets, the reasons for prescribed doses were poorly understood. Dietary knowledge, while influenced by ANM counselling and local beliefs, remained superficial, women commonly mentioned eating fruits, milk, and vegetables but were unable to connect these foods to anaemia prevention. One woman stated, “*ANM gave me good ideas when I asked he*r,” suggesting information was obtained only reactively. Some positive Hygiene practices like washing vegetables before cooking were reported. However, tools like the Mother and Child Protection (MCP) card were under-utilized and misunderstood, with one woman remarking, “*Nothing is written in the vaccination card; it's only used for vaccines,*” indicating a missed opportunity for communication and record-based counselling.

#### 4.1.2 Opportunity: access, availability and support systems

While transport options to health facilities were generally available, economic barriers and gendered dependence often restricted timely care. As one woman explained “*Vehicles run.* *but when my husband is not working, I don't prefer to use it unless it is very important for my unborn child.”* This reflects a willingness to prioritize health when necessary but also reveals economic barriers and dependence on male family members. Irregular access to IFA supplements and frequent stock-outs were widely reported. Women received partial supplies or were advised to seek IV iron sucrose doses privately. One woman shared, “*Only one injection was available at the facility; I took the remaining five from a nearby doctor.”* Counselling from frontline workers was inconsistent, and postnatal guidance was almost entirely absent. Anganwadi meetings often lacked follow-up, and women criticized the poor quality of supplementary food from ICDS centres: “*Only one packet (milk powder) was given, and it was lumpy  … didn't like it”.*

##### 4.1.2.1 Information, education and communication (IEC) activities

IEC activities were sparse or ineffective. A lactating mother recalled, “*No one said anything after delivery  … I want to know what is good for my child and me, but no one tells or knows the correct things”.* The lack of counselling created confusion about anaemia management. Additionally, the study team observed that such service delivery gaps reinforced dependence on private providers and weakened trust in public services.

#### 4.1.3 Motivation: beliefs, attitudes, and behavioural drivers

Motivational barriers largely stem from physical side-effects, cultural beliefs, and limited perceived benefits. Nausea and vomiting were commonly attributed to IFA tablets, with one pregnant woman admitting, “*The small red pill made me vomit  … I prefer throwing it than consuming it”*. This not only affected adherence but created negative associations with supplementation.

Cultural beliefs also influence behaviors. Many women a reduced food consumption in the last trimester, fearing large babies and difficult deliveries. As one explained, “*In the last month I reduced eating; if the baby grows big, it will be hard to deliver”*. Family members including mother-in-law or older women often influenced dietary and medication choices. A lactating mother shared, “*My mother told me not to eat sour food; it causes the stitches to get infected  … so I listened to her”.*

Despite these barriers, some women exhibited intrinsic motivation to attend antenatal check-ups and facility visits, particularly when they received timely investigations or reassuring feedback “*The doctor checked my eyes and tongue and said haemoglobin is fine  … all these made me feel good”.* However, this motivation was frequently dampened by rude behaviour and lack of follow-up, highlighting how interpersonal dynamics strongly affected compliance.

#### 4.1.4 Emergent health-system factors (*inductive themes*)

Beyond individual and behavioural determinants, several systemic challenges emerged inductively. Women cited poor provider communication, long waiting times, and inconsistent service delivery as deterrents to continued engagement. One woman remarked, “*There was a long queue; my number never came”.* Participants repeatedly referenced stock-outs, irregular counselling sessions, and missed opportunities for postnatal follow-up, revealing gaps in continuity of care. Social determinants, particularly financial constraints and limited household decision-making power were also observed as a hinderance to consistent healthcare utilization.

### 4.2 Qualitative findings from healthcare providers and community health staff

This analysis revealed how capability, opportunity, and motivation interacted to influence providers' practices and service delivery within the Anaemia Mukt Bharat (AMB) program. Additional inductive themes related to program fragility, trust deficits, and gendered constraints also emerged beyond the core COM-B domains (Table 5).

**Table 5:** Qualitative findings using mixed analysis i.e., inductive and deductive (COM-B) model to summarize the findings from healthcare providers and community health staff.

Theme	Subtheme	Verbatim
Capability	Knowledge of anaemia and its classification	*Blood deficiency is called haemoglobin deficiency* (**ANM**)
		*“If Hb is below 7 gm, the life of both mother and child may be at risk and condition is called anemia”* (**ASHA)**
		*“If Hb is below 6 gm, it is considered severe anemia.”(**Staff Nurse**)*
	Understanding of dietary needs in pregnancy	*“Pregnant women should eat leafy green vegetables and jaggery with roasted chickpeas*.*”* **(ANM)**
		*“Leafy vegetables, seasonal fruits, and milk or yogurt should be consumed.”* (**ASHA)**
		*“Whatever is cooked at home, they should eat it in small portions throughout the day*.*”* (**Staff Nurse**)
	Awareness about ANC, its schedule and importance	*“Four ANC check-ups should be done, and iron and calcium tablets should be taken*.*”* (**ASHA)**
		*“First ANC visit should be by 12 weeks, second by 24 weeks, third by 28 weeks, and fourth by 36 weeks*.*”* (**Staff Nurse)**
		*“There should be at least one check-up per trimester.” (**MO)***
	Knowledge about treatment protocols	*“If Hb is below 9 gm, one IFA tablet daily; if below 8 gm, two tablets daily; and if below 7 gm, then iron sucrose injections.”* (**Staff Nurse)**
		*“Iron sucrose should be given in 7 doses on alternate days*.*”* (**MO)**
		*“If Hb is low during delivery, we perform blood transfusion.”* (**ANM**)
	Use of digital tools for reporting	*“We enter data in the ANMOL app and also maintain it in registers*.*”* **(ANM)**
		*“We give monthly data to the Information Assistant (IA), and it is recorded online.”* (**Staff Nurse)**
Opportunity	Access to government ANC services	*“On the 9th, 10th, and 24th, of every month women are called and given nutritional supplements.”* (**ANM)**
		*On the 9^th^ of every month, check-ups are done, doctors come, and juice, biscuits are distributed * … … *I feel the expecting mothers are benefited greatly by these initiatives (**ASHA)***
		*Camps for pregnant women are held every month on the 9th, 10th, and 23rd* … *. this help detecting the high risk cases”* (**MO)**
	Availability of IFA/calcium	*“IFA tablets are often unavailable, and the syrup that comes is meant for children.”* (**ANM)**
		*“Sometimes tablets are available, sometimes not..*.*patients are told to ask ASHA.” (***Staff Nurse)**
		*“IFA stock is available for 8 months of the year, and not available for the remaining 4*.*”* (**MO)**
	Incentives and reporting systems	*“Earlier, ASHAs received*₹*125 for every ANC check- up.”* (**ASHA)**
		*We submit monthly reports to IA; all records are maintained in the register* … *. this way the system is robust and systematic.’* (**Staff Nurse**)
		*ANMs keep the records in registers, and the IA collects the data*.*”* (**MO**)
	VHND and counselling platforms	*“We conduct counseling during Poshan Pakhwada (nutrition day celebration) and (Godbharai ceremonies) celebrating pregnancy status of women at health facilities).”* (**CDPO)**
		*“During VHND, we talk about iron-rich diets and ANC check-ups.”* (**Staff Nurse)**
		*“On Poshan Diwas (Nutrition-Day), counseling is held everywhere, and we use charts to explain.”* (**ASHA)**
Motivation	Perceived side-effects of IFA	*I don't think the beneficiaries like the taste and aftereffects for these medicines* … *. I, myself, feel nauseous and don't feel like taking the tablets.’* (**ASHA)**
		*“Most of the beneficiaries deny tablets as they have abdomen pain and nausea* … … . *If IFA tablets don't suit the patient, we provide syrup instead.”* (**Staff Nurse)**
		*“Women stop taking the tablets because of nausea.”* **(MO)**
	Cultural norms and compliance	*In this community, some people have money and among them some women say prefer going to private doctors and don't attend meetings*.’ (**ASHA)**
		*Family members don't allow women to go alone or don't give them time*.’ (**Staff Nurse)**
		*Newly married daughters-in-law are not allowed to go out for ANC* … . *they will be needed to be accompanied by someone from family* … . *So, if no one free, new expecting mothers don't visit the sessions.’* (**MO)**
	Self-driven behavior and prioritization	*Women who are compliant and don't have any side- effects* … *ask for extra tablets themselves.’* (**Staff Nurse**)
		*Some patients say food will fix everything; they don't want medicine * … … *but they ask what food should be consumed for optimal health’* (**MO)**
Program Fragility	Implementation gaps	The AMB program starts first, and supplies arrive later … . this will never help us to give medicines to beneficiaries on time.’ (ASHA)
	IFA stock inconsistency	*“*IFA tablet stock is never consistent.*”* (MO)
Missed opportunities	Delay in ANC registration	*Most of the time the ANC registration is delayed, and one major reason is confirmation of pregnancy, gets delayed due to issues like no radiologist for sonography and referral of patients to private facilities* … *. this results into loosing suspected pregnancy and delivering them required supplementation at time*’ (**Staff Nurse**)
	Diagnostic bottlenecks	*We sometimes don't have supply of required equipment for diagnosis of anaemia, so even if women are visiting ANC at right time* … *. We miss out on diagnosing them (**Staff nurse)** -For confirmation of anaemia we are referring women to higher facilities as no supplies for required equipment since few days (**ANM)***
	Missed opportunities for preconception & postpartum	*“Hb test should be done even before conception, and folic acid should be started early*.*”* (**MO)**
		*After 42 days, no one provides counseling* … *no one checks on the mother after that.”* (**Staff Nurse)**
Program Fragility and Systemic Challenges (Inductive Themes)		
Provider– Patient Disconnect and Trust Deficits	Health worker attitude	*“People don't like the way government hospital staff counsels' beneficiaries and handles them*.*”* (**ASHA**)
	Depleting patient trust	*“Sometimes the staff is rude, so women prefer going to private hospitals* … . *as they think they will be treated well and don't trust the government centers at all”* (**CDPO)**
Autonomy	Gender and power dynamics	*“Here, the value of a woman is no more than that of a buffalo in the household* … … . *how can we expect women will take care of their health”* (**MO)**
		*Decisions in the house are taken by the husband or mother-in-law; the woman doesn't have a say*.’ (ASHA)

#### 4.2.1 Capability: knowledge, skills, and technical proficiency

Across cadres- ASHAs, ANMs, staff nurses, and medical officers- providers demonstrated a strong foundational understanding of anaemia and its management. They consistently defined anaemia as “blood deficiency” and were able to classify its severity. As an ASHA noted, “If Hb is below 7 gm, the life of both mother and child may be at risk and condition is called anemia.” A staff nurse reinforced this, adding, “If Hb is below 6 gm, it is considered severe anemia”.

Providers also articulated accurate knowledge of dietary management during pregnancy, recommending “leafy green vegetables, jaggery with roasted chickpeas, milk, and fruits.” Awareness of antenatal care (ANC) scheduling was high, with staff clearly citing standard visit timelines: “First ANC visit should be by 12 weeks, second by 24 weeks*  …* ” Providers also had clarity on AMB treatment protocols. Staff explained when to start iron sucrose and how many doses to give, such as “Iron sucrose should be given in 7 doses on alternate days.” Digital proficiency was also evident, ANMs confidently reported using the ANMOL app and routinely submitting reports to Information Assistants. However, despite this solid knowledge base, a few respondents revealed gaps in refresher training and expressed uncertainty about updated AMB guidelines, indicating a need for continuous capacity building.

#### 4.2.2 Opportunity: enabling systems and operational barriers

Health system context provided multiple opportunities and shaped providers' ability to deliver quality AMB services. Government-supported ANC platforms such as the Pradhan Mantri Surakshit Matritva Abhiyan (PMSMA) and Village Health and Nutrition Days (VHNDs) were viewed positively by staff as important channels for service delivery and community outreach. An ASHA shared, “On the 9th of every month, check-ups are done, doctors come, and juice, biscuits are distributed.  I feel the expecting mothers benefit greatly”. However, despite these structured platforms, several operational barriers persisted. One of the most frequently reported issues was stock inconsistency. As a Medical Officer admitted, “IFA tablet stock is never consistent,” and an ANM added, “The program starts first, and supplies arrive later*  …*  this will never help us give medicines to beneficiaries on time”. Facilities often had only partial IFA supplies, and ANC tests were sometimes missed due to non-functional Hb devices or lack of testing strips. These supply-chain and diagnostic limitations constrained the ability of frontline staff to provide continuous care. Providers also expressed dissatisfaction with weak follow-up systems and gaps in postnatal counselling. One staff nurse noted, “After 42 days, no one provides counselling*  …* no one checks on the mother after that”. In addition, the quality of dietary supplements distributed through Integrated childhood development scheme (ICDS) centres was often poor. As one staff member recalled, “Only one packet (milk powder) was given, and it was lumpy*  …* didn't like it”. Although counselling activities were conducted during special events such as Poshan Pakhwada and Godbharai ceremonies, their effectiveness varied due to limited IEC resources and irregular follow-up.

#### 4.2.3 Motivation: attitudes, beliefs, and cultural contexts

Healthcare providers consistently cited perceived side effects of IFA as a major deterrent for compliance. “Most beneficiaries deny tablets as they have abdomen pain and nausea,” shared a staff nurse. An ASHA candidly admitted, “I, myself, feel nauseous and don't feel like taking the tablets”. Such shared experiences shaped how they counselled women and reflected a need for empathetic, experience-based communication strategies. Cultural norms and family also affected service uptake. Staff observed that newly married daughters-in-law were often restricted from attending ANC visits unless accompanied by a family member. A medical officer remarked “Newly married daughters-in-law are not allowed to go out for ANC  *…* if no one is free to accompany them, they don't visit the sessions,” Nevertheless, providers reported that some women were self-motivated and proactive, requesting extra tablets or seeking dietary advice. As one nurse noted, “Women who are compliant and don't have side effects ask for extra tablets themselves”. These instances underscore the potential for positive deviance and empowerment-based counselling to improve adherence.

#### 4.2.4 Program fragility and systemic challenges (inductive themes)

Beyond individual-level determinants, several structural issues emerged inductively. Providers pointed to implementation gaps and fragile program logistics, where “the AMB program starts first, and supplies arrive later”. Diagnostic limitations were common like delays in obtaining Hb-testing equipment or strips led to missed diagnoses. “We sometimes don't have the supply of required equipment for diagnosis*  …* so even if women come at the right time, we miss out on detecting anaemia,” explained a staff nurse. Similarly, missed opportunities during pre-conception and postnatal care were widespread. A medical officer noted, “Hb test should be done even before conception, and folic acid should be started early”.

## 5 Discussion

This formative research, conducted as Phase-I of an implementation research project, highlights critical implementation gaps impeding effective implementation of the Anemia Mukt Bharat (AMB) program in Palwal district, Haryana. The findings underscore a significant gap between the intended coverage and actual service delivery and compliance outcomes, reflecting persistent health system inefficiencies and socio-cultural barriers despite longstanding policy initiatives.

Consistent with earlier studies conducted in similar LMIC contexts, our findings confirm that health workers possess fundamental knowledge about anemia management protocols, dietary recommendations, and ANC scheduling (24, 25). However, despite adequate knowledge among healthcare providers, service provision remains suboptimal due to irregular availability of IFA stocks, inadequate diagnostic tools, and fragmented service delivery systems. Similar supply chain disruptions have been widely reported across India and other South Asian regions (26) (27, 28), highlighting systemic barriers that transcend individual healthcare providers' capacities. Our results, indicating only 67% of women receiving any IFA tablets and a mere 5% compliance with anemia-specific dosing guidelines. These findings corroborate national-level analyses reflecting low adherence and insufficient supplementation (29).

Women's limited capability was apparent through superficial understanding of anemia, misconceptions about diagnostic procedures, and inadequate use of health records like MCP cards. These findings resonate with previous research emphasizing the necessity of enhanced SBCC strategies to foster deeper health literacy among beneficiaries (16, 30). Furthermore, motivational barriers due to IFA-induced gastrointestinal side-effects are biologically plausible and widely documented (31). Side-effects including nausea, vomiting, and gastrointestinal distress substantially impede compliance, necessitating strategies such as counselling, divided doses, or alternative formulations like enteric-coated tablets (32). These findings also suggest that perceived side effects may lead to both underestimation of true compliance (due to recall or social desirability bias) and overestimation of coverage when based only on reported receipt rather than actual consumption, a limitation acknowledged in similar field settings.

Opportunities to access healthcare were theoretically sufficient (e.g., VHND, PMSMA), yet practically inadequate due to irregular scheduling, poor attendance, and compromised quality of dietary supplementation. Similar gaps in public health outreach programs have been observed previously, limiting the effectiveness of nutrition and maternal health interventions across rural India (33). Our findings highlight that socio-economic constraints and household responsibilities also severely limit women's autonomy and healthcare access; a pattern consistent with other studies conducted in similar socio-cultural environments in South Asia (34, 35).

A particularly critical emergent theme in this study was the role of provider-patient trust deficits and healthcare worker attitudes. Negative experiences at government facilities propelled women towards private care, compromising the effectiveness of public programs. Restoring trust through compassionate communication, respectful maternity care, and consistent service quality are essential for improving engagement and adherence. Similar trust issues have previously been noted as significant barriers to maternal healthcare utilization in India and other LMICs (36, 37). Additionally, patriarchal family structures and cultural norms restricting women's independent mobility emerged as substantial barriers, reinforcing findings from previous studies that underscore the importance of family-centered approaches to maternal health interventions (35).

Biologically, anemia during pregnancy substantially increases risks for maternal morbidity, preterm births, low birth weight, and cognitive impairment in offspring, underscoring the urgency of addressing these implementation challenges (38, 39). Effective delivery and adherence to IFA supplementation can significantly improve maternal and neonatal outcomes, reinforcing the critical need for robust, system-level interventions and improved community engagement strategies.

### 5.1 Implications

This study offers critical insights for beneficiaries by highlighting the necessity of improved health literacy and culturally sensitive behavior to change communication. For healthcare providers, findings underscore the importance of regular refresher training, effective counseling techniques, and addressing interpersonal skills to improve trust and patient-provider relationships. Health systems and policy implications include prioritizing supply chain robustness, ensuring diagnostic readiness, and integrating comprehensive preconception and postpartum care. In terms of sustainability and scalability, the AMB implementation model could be strengthened through institutionalized refresher trainings, decentralized procurement systems, and integration with existing maternal–child health platforms such as PMSMA and VHND to ensure continuity. At the policy level, findings call for revising existing program strategies to include systematic community engagement and sustained multi-level monitoring mechanisms to ensure compliance and effectiveness. The insights generated are contextually rooted but methodologically adaptable, providing a replicable model that can be generalized to other low-performing districts in Haryana and similar settings across India where socio-cultural and system-level determinants are comparable.

Insights from this formative phase will guide the optimization and implementation of delivery model in Phase-II. Implementation strategies will prioritize regular and reliable stock provision, strengthened diagnostic facilities, continuous and supportive counseling for managing side-effects, community-based behavior change initiatives, and structured family engagement programs. Moreover, addressing provider attitudes and enhancing healthcare system accountability will be integral to bridging the gaps identified in Phase-I, ultimately aiming to significantly enhance effective coverage and compliance within the AMB program.

### 5.2 Strengths and limitations

This study utilized a robust mixed-methods design, combining quantitative and qualitative data to provide comprehensive insights into implementation challenges at multiple levels (health systems, providers, and beneficiaries). Employing the COM-B behavioural framework allowed structured identification of capability, opportunity, and motivational factors influencing behaviour. Additionally, integrating diverse stakeholders, including beneficiaries, community healthcare providers, and health system staff, strengthened the depth and practical applicability of findings. A limitation was the cross-sectional nature of the baseline quantitative assessment, whichmrestricted causal interpretations. The purposive sampling of clusters and participants might have introduced selection bias, limiting generalizability. Additionally, recall bias in reporting IFA tablet consumption and ANC visits by pregnant women could have affected the accuracy of self-reported compliance data. This may have resulted in slight overestimation of program coverage and underestimation of adherence rates. Furthermore, the service provision was only assessed at government facilities, as private-sector IFA supplement practices were not captured, possibly leading to information bias.

## 6 Conclusion

This formative research underscores significant implementation gaps in the Anaemia Mukt Bharat (AMB) program, particularly concerning supply chain inconsistencies, suboptimal adherence to clinical guidelines, inadequate beneficiary counselling, and restrictive sociocultural norms. Effective AMB implementation requires addressing these multifaceted barriers through systematic improvements in health system logistics, tailored social behavior change communication, enhanced training of healthcare providers, and strengthened community engagement. These findings lay a foundation for developing optimized implementation strategies in subsequent phases, aiming for substantial reductions in anaemia prevalence among pregnant women (40).

## Data Availability

The raw data supporting the conclusions of this article will be made available by the authors, without undue reservation.
