# Supplementary material for: Implementation challenges of India's national anaemia reduction program among pregnant women: insights from mixed-methods implementation research
Source: Front Glob Womens Health. 2026 Apr 13;7:1695442. doi: 10.3389/fgwh.2026.1695442 (PMC13111238; doi:10.3389/fgwh.2026.1695442)

**Supplementary Material**

**Figure 1:** Status of anaemia of the pregnant women in the catchment area who had Hb status available and had received IFA


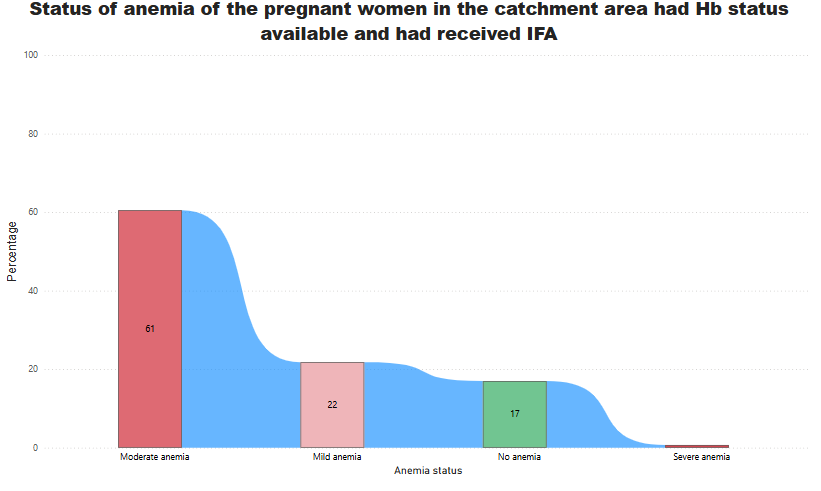

Supplement: Supplementary file 1 [file Supplementaryfile1.docx]
